# Missing School Days Following Sport-Related Concussion in High School Athletes

**DOI:** 10.1001/jamanetworkopen.2024.40264

**Published:** 2024-10-18

**Authors:** Tracey Covassin, Alyssa M. Pollard-McGrandy, Lilian A. Klein, Douglas J. Wiebe, Abigail C. Bretzin

**Affiliations:** 1 Michigan State University, East Lansing; 2 University of Michigan, Ann Arbor

## Abstract

**Importance:**

Sport-related concussions (SRC) can significantly impact students’ attendance and academic performance, highlighting the importance of gradual return-to-learn protocols for recovery. Understanding the association between missed school days and recovery, especially across genders, is vital for effective concussion management in high school athletes.

**Objectives:**

To describe missed school days in high school athletes with SRC and to determine associations between missed school days and authorized clearance to return to competition; secondarily, to determine whether school days missed differed by gender.

**Design, Setting, and Participants:**

Cohort study among student athletes participating in athletics sponsored by the Michigan High School Athletic Association (MHSAA) during academic years 2015 to 2023. SRC was a participant eligibility criterion and was defined as a head injury during participation in a MHSAA-sanctioned event. Data were analyzed from August 2015 to June 2023.

**Exposures:**

Primary exposure was missed school days; additional exposures included gender, year of season, concussion history, event type, sport level, contact level, and athletic trainer involvement.

**Main Outcome and Measure:**

The primary outcome was time to full unrestricted clearance following SRC.

**Results:**

The analysis included a total of 20 934 individuals with SRC (13 869 boys [66.25%]). Gender (χ^2^_3_ = 167.40; *P* < .001), season (χ^2^_21_ = 57.41; *P* < .001), event type (χ^2^_3_ = 99.29; *P* < .001), sport level (χ^2^_9_ = 68.15; *P* < .001), contact level (χ^2^_6_ = 56.73; *P* < .001), and initial evaluation (χ^2^_3_ = 147.13; *P* < .001) were independently associated with missed school days. Athletes took a median (IQR) of 11 (7-16) days to return to full unrestricted clearance. Relative to no missed school days, there was a significant mean increase of 57% (incidence rate ratio, 1.57; 95% CI, 1.52-1.62) in time to full unrestricted clearance for 3 or more missed school days. The estimated mean days to return to sport were 12.15 (95% CI, 12.00-12.30) for 0 missed school days, 12.68 (95% CI, 12.39-12.96) for 1 missed school day, 15.47 (95% CI, 15.06-15.87) for 2 missed school days, and 19.08 (95% CI, 18.55-19.62) for 3 or more missed school days.

**Conclusions and Relevance:**

This cohort study found that high school athletes typically missed 2 or fewer school days after SRC, suggesting that concussion management teams adhered to current recommendations of 24 to 48 hours of initial rest. The dose association, with more missed school days associated with slower return to play, suggests athletes’ missing more school days (eg, ≥3) may be associated with severity of concussion and warrant additional support to return to school and/or sport.

## Key Points

**Question:**

How many missed school days typically occur in high school boys and girls with sport-related concussion, and what is the association between missed school days and authorized clearance to return to competition?

**Findings:**

In this cohort study, including 20 934 high school athletes with sport-related concussion, students typically missed 2 or fewer school days and took a median of 11 days to return to full medical clearance. A significant association was observed, with more missed school days associated with slower time to return to sport.

**Meaning:**

This study suggests that current concussion management guidelines that include an initial rest period are followed and that missing more school days may be associated with severity of concussion and warrant additional supports to return to school and sport.

## Teaser

This cohort study evaluates the association between missed school days in high school athletes with sport-related concussion and authorized clearance to return to competition.

## Introduction

Between 1.1 to 1.9 million sport and recreation–related concussions occur annually among children and adolescents in the US.^1^ Among high school and collegiate athletes, the most prevalent symptoms following a sport-related concussion (SRC) include headaches, dizziness, difficulty concentrating, confusion, and visual problems and/or sensitivity to light.^2,3^ These symptoms can significantly impede adolescents’ ability to attend school and can detrimentally affect their academic performance. Hence, the return-to-learn (RTL) process holds paramount importance following an SRC, especially for adolescent students. The most recent Concussion in Sport Group Consensus Statement recommends a brief 24 to 48 hour rest period, during which students can engage in light activities, such as 5 to 15 minutes of screen time or reading, that do not exacerbate symptoms beyond mild discomfort.^4^ Following this initial rest period, students should gradually reintegrate into school activities, starting with homework and increased reading time, then transitioning to part-time school attendance before returning full-time.^4^ Recent research indicates that prolonged strict rest hinders recovery, whereas engaging in early, gradual cognitive and physical activities of moderate intensity enhances RTL.^5,6,7^

There are conflicting findings regarding the relationship between missed school days and concussion recovery. A recent meta-analysis^8^ revealed that on average, it took 8.3 days for athletes to fully RTL, with the majority of athletes across all age groups returning to full academic activities without needing academic support by day 10. Previous research suggests high school and collegiate students take 5 to 7 days to return to school,^9,10^ while other researchers reported that high school athletes only missed on average 1 day of school.^11^ Wasserman and colleagues found 24% of high school and collegiate students had not returned to school after 1 week.^10^ However, some hypothesize that extended absences from school and other daily activities following a concussion could impede recovery. Returning to school promptly may correlate with reduced symptom severity and, consequently, expedited recovery.^6,12,13^ Specifically, enforcing strict rest for 5 days (ie, abstaining from school and physical activity) compared with 1 to 2 days led to a slower recovery of symptoms.^6^ These findings suggest variability in recovery times, but they are complicated by the inclusion of both high school and collegiate populations in many studies, which may obscure specific trends within each group. Additionally, a randomized clinical trial by Thomas et al,^6^ which provided valuable insights into concussion recovery, focused on a broader age range (11-22 years) and emergency department settings. This may not fully capture the unique academic and social pressures faced by high school students compared with collegiate athletes. For instance, high school students often experience different academic and extracurricular demands, which could influence their recovery trajectory differently from collegiate athletes. Consequently, the applicability of the findings by Thomas et al^6^ to the high school population specifically may be limited. Given these considerations, it is crucial to investigate whether high school students who miss more school days have longer recovery periods from concussions, as existing studies may not adequately address these differences.

Studies show that girls are more susceptible to concussions relative to boys in similar activities.^14,15^ Recent research at the collegiate level indicates that boys and girls have comparable time loss for returning to sports.^16,17,18,19,20^ However, some studies suggest that at the high school level, girls require more time to recover compared with boys.^21^ Currently, there is insufficient research comparing missed school days at the high school level between boys and girls. Bretzin and colleagues^21^ reported no significant differences in the average number of missed school days between boys and girls. Conversely, in girls’ sports, athletes tend to experience longer durations of symptoms and require more time to return to academic activities compared with boys’ sports.^22^ In addition, other factors, including concussion history, the level of participation (ie, freshman, junior varsity, or varsity), sport contact level (ie, collision and/or contact, limited contact, or noncontact), academic year of concussion, more recent concussions due to heightened awareness and/or lingering effects of the pandemic and at-home school options, or even the personnel involved in the initial evaluation may influence time missed from school,^23,24,25^ motivating us to evaluate time missed from school in a large cohort of high school athletes. Therefore, the purpose of this study was to describe missed school days in high school athletes with SRC and determine the association between missed school days and authorized clearance to return to competition. Secondarily, we aim to determine if girls missed more school days than boys in this adolescent sample. We hypothesized that adolescents with more missed school days would take longer to return to full medical clearance, and girls would miss more school days than boys.

## Methods

### Study Design, Schools, and Participants

**Figure 1.**
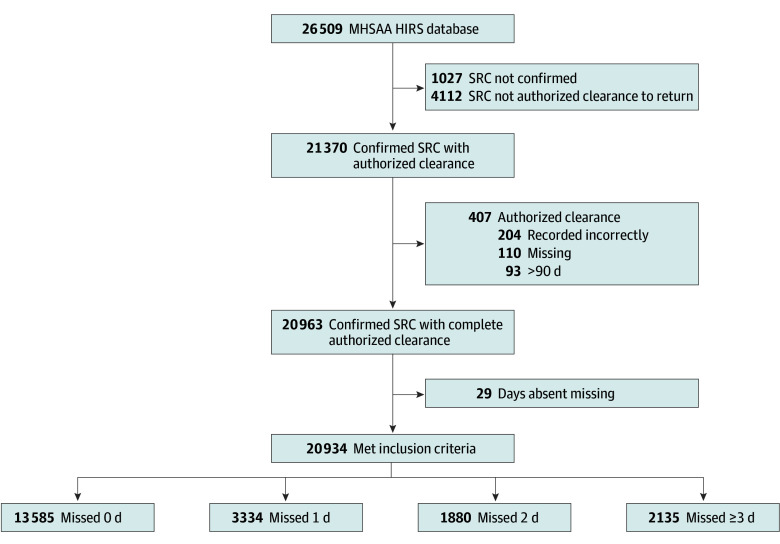
Sport-Related Concussion (SRC) Cases in the Michigan High School Athletic Association (MHSAA) Head Injury Reporting System (HIRS) Databases Included in Final Analyses

The present research was exempt from review by the Michigan State University institutional review board and informed consent was not required because data were deidentified. This study followed the Strengthening the Reporting of Observational Studies in Epidemiology (STROBE) reporting guideline for observational studies.^26^ A cohort study conducted among approximately 750 high schools affiliated with the Michigan High School Athletic Association (MHSAA) consistently records incidents of head injuries through a surveillance system established in the 2015 to 2016 academic year. Study participants included all documented SRC cases across an 8-year period (2015/2016-2022/2023) and all MHSAA high school athletes participating across the association (26 509 individuals). Sport contact level was classified by the American Academy of Pediatrics into collision and/or contact, limited contact, and noncontact.^27^ SRC cases were excluded if the diagnosis was not confirmed, authorized clearance data were not recorded, authorized clearance data were recorded incorrectly (eg, clearance date occurred before incident SRC date), authorized clearance was 90 or more days after injury, or missed school days was missing (Figure 1).

### Operational Definitions

#### Sport-Related Concussion

SRC was defined as a head injury during participation in an MHSAA-sanctioned in-season practice, scrimmage, or game, including an MHSAA-provided postseason tournament. Athletes were required to refrain from further activity if they displayed signs (ie, loss of consciousness), symptoms (ie, headache), or behaviors (ie, confusion) consistent with SRC. As outlined in the MHSAA concussion protocol, all SRCs must receive diagnosis from 1 of 4 clinician types (eg, doctor of osteopathic medicine [DO], medical doctor [MD], nurse practitioner [NP], or physician assistant [PA]). We only included confirmed SRC cases in the analyses.

#### Missed School Days

Missed school days were defined as number of days absent from school following the SRC incident. Missed school days were either self-reported by the student to the athletic trainer, coach, or administrator or accessed through school attendance records. Academic accommodations or supports are not currently recorded in the MHSAA database.

#### Full Unrestricted Clearance

Full unrestricted clearance was established as the time between the SRC incident until the day the athlete received complete medical clearance from 1 of 4 authorized health care clinician types (ie, MD, DO, NP, or PA). The MHSAA specifically states, “The full unrestricted clearance must be in writing and must be unconditional. It is not sufficient that the MD, DO, PA or NP has approved the student to begin a return-to-play progression. The clinician must approve the student’s return to unrestricted activity. Individual schools, districts and leagues may have more stringent requirements and protocols including but not limited to mandatory periods of inactivity, screening and postconcussion testing before or after the written clearance for return to activity.”^28^

### Procedures

The MHSAA developed the Head Injury Reporting System (HIRS) specifically for use by athletic trainers, coaches, and administrators. Access to the MHSAA login is restricted to identified users, who are mandated to input information regarding participants and head injuries. Given the diverse personnel and resources available at each member school, the MHSAA does not stipulate who is responsible for entering the information at each institution, leaving this task to the discretion of individual schools. In schools where athletes experienced SRC, reporting was required in 2 phases with a linked unique 7-digit study ID: (1) an initial head injury report that included the participant’s grade, gender, sport, date and time of injury, and injury circumstances (ie, event type and mechanism), and (2) a subsequent follow-up report. The follow-up report entailed circumstances of recovery (ie, date of full medical clearance, medical professional responsible for authorizing a return, and number of missed school days).

MHSAA staff continuously monitors HIRS data for inaccuracies and missing information, and they are the sole individuals authorized to adjust SRC reports. To ensure the thoroughness of data input, MHSAA staff regularly scrutinized follow-up reports on a weekly basis. Every 2 to 3 weeks, MHSAA staff sent emails to each school that submitted an initial report, reminding them to submit a final follow-up report. At the conclusion of each sports season, schools without any head injury reports were required to document this absence in the HIRS. Follow-up procedures persist throughout each season for schools that have either not reported any concussions or have reported 0 concussions until data have been received from no less than 99% of MHSAA member high schools.

### Statistical Analysis

We used descriptive characteristics to describe our study sample, presenting aggregate data in frequencies and percentages for categorical variables and medians and IQRs for continuous variables. To describe missed school days in our sample, we provided descriptive statistics for the number of missed school days (eg, 0, 1, 2, ≥3) by each of our demographic variables. We then tested the association with missed school day category between boys and girls, concussion history, sport level, contact level, season, having an athletic trainer in the initial concussion evaluation, and event type with 2-sided χ^2^ analysis (Bonferroni corrected *P* < .007). Last, we presented our time to return to sport outcome by missed school days with Kaplan-Meier curves. Based on nonnormal distribution of data, we conducted a final multivariable Poisson regression with robust standard errors to test the association between missed school days and time to return to sport, and estimate the average days to recovery based on the number of missed school days. We included variables previously determined to be associated with return to sport time.^23^ The final regression was determined significant if the 95% CI excluded 1. All analyses were completed in Stata version 17 (StataCorp). Data were analyzed from August 2015 to June 2023.

## Results

Our analysis includes a total of 20 934 SRC cases (13 869 boys [66.25%]). The majority of SRC cases (13 677 cases [65.33%]) occurred in competition, and over half of SRC cases were sustained by varsity athletes (12 110 cases [57.85%]). Separately, a majority of SRC cases had no concussion history (17 739 cases [84.74%]). Almost three-quarters of SRCs were evaluated initially by an athletic trainer (15 365 cases [73.40%]). We present descriptive statistics in Table 1.

**Table 1.** Descriptive Characteristics of Sport-Related Concussion Cases Missing 0, 1, 2, or 3 or More School Days

Demographic	Participants, No. (%)					χ^2 ^(degrees of freedom)	*P* value
	Overall (N = 20 934)	0 d (n = 13 585)	1 d (n = 3334 )	2 d (n = 1880)	≥3 d (n = 2135)		
Season							
2015-2016	3546 (16.94)	2323 (65.51)	523 (14.75)	329 (9.28)	371 (10.46)	57.41 (21)	<.001
2016-2017	3320 (15.86)	2161 (65.09)	509 (15.33)	300 (9.04)	350 (10.54)		
2017-2018	2870 (13.71)	1844 (64.25)	480 (16.72)	274 (9.55)	272 (9.48)		
2018-2019	2951 (14.1)	1974 (66.89)	472 (15.99)	232 (7.86)	273 (9.25)		
2019-2020	2335 (11.15)	1523 (65.22)	382 (16.36)	212 (9.08)	218 (9.34)		
2020-2021	1228 (5.87)	813 (66.21)	161 (13.11)	91 (7.41)	163 (13.27)		
2021-2022	2147 (10.26)	1332 (62.04)	354 (16.49)	214 (9.97)	247 (11.5)		
2022-2023	2537 (12.12)	1615 (63.66)	453 (17.86)	228 (8.99)	241 (9.5)		
Gender							
Girls	7065 (33.75)	4170 (59.02)	1284 (18.17)	784 (11.1)	827 (11.71)	167.40 (3)	<.001
Boys	13 869 (66.25)	9415 (67.89)	2050 (14.78)	1096 (7.9)	1308 (9.43)		
Concussion history							
0	17 739 (84.74)	11 567 (65.21)	2801 (15.79)	1573 (8.87)	1798 (10.14)	8.80 (6)	.19
1	2427 (11.59)	1553 (63.99)	396 (16.32)	224 (9.23)	254 (10.47)		
≥2	768 (3.67)	465 (60.55)	137 (17.84)	83 (10.81)	83 (10.81)		
Event type							
Practice	7257 (34.67)	5033 (69.35)	978 (13.48)	585 (8.06)	661 (9.11)	99.29 (3)	<.001
Competition	13 677 (65.33)	8552 (62.53)	2356 (17.23)	1295 (9.47)	1474 (10.78)		
Sport level							
Middle school	102 (0.49)	60 (58.82)	24 (23.53)	9 (8.82)	9 (8.82)	68.15 (9)	<.001
Freshman	2177 (10.4)	1532 (70.37)	313 (14.38)	184 (8.45)	148 (6.8)		
Junior varsity	6545 (31.26)	4291 (65.56)	1073 (16.39)	571 (8.72)	610 (9.32)		
Varsity	12 110 (57.85)	7702 (63.6)	1924 (15.89)	1116 (9.22)	1368 (11.3)		
Contact level							
Collision/contact	18 432 (88.05)	12 107 (65.68)	2875 (15.6)	1619 (8.78)	1831 (9.93)	56.73 (6)	<.001
Limited contact	2042 (9.75)	1174 (57.49)	383 (18.76)	228 (11.17)	257 (12.59)		
Noncontact	460 (2.2)	304 (66.09)	76 (16.52)	33 (7.17)	47 (10.22)		
First evaluation by athletic trainer							
Yes	15 365 (73.4)	10 321 (67.17)	2307 (15.01)	1335 (8.69)	1402 (9.12)	147.13 (3)	<.001
No	5569 (26.6)	3264 (58.61)	1027 (18.44)	545 (9.79)	733 (13.16)		

### Missed School

Overall, 3334 boys and girls (15.9%) missed 1 day, 1880 (9.0%) missed 2 days, and 2135 (10.2%) missed 3 or more days of school following SRC. Table 1 presents missed school day category by each of our demographic variables. We found that gender was significantly associated with missed school days, as 4170 girls (59.0%) and 9415 boys (67.9%) missed 0 school days after SRC. The number of missed school days was independently associated with the season (χ^2^_21_ = 57.41; *P* < .001), gender (χ^2^_3_ = 167.40; *P* < .001), event type (χ^2^_3_ = 99.29; *P* < .001), sport level (χ^2^_9_ = 68.15; *P* < .001), contact level (χ^2^_6_ = 56.73; *P* < .001), and having an athletic trainer in the initial SRC evaluation (χ^2^_3_ = 147.13; *P* < .001).

### Unrestricted Return to Sport

**Figure 2.**
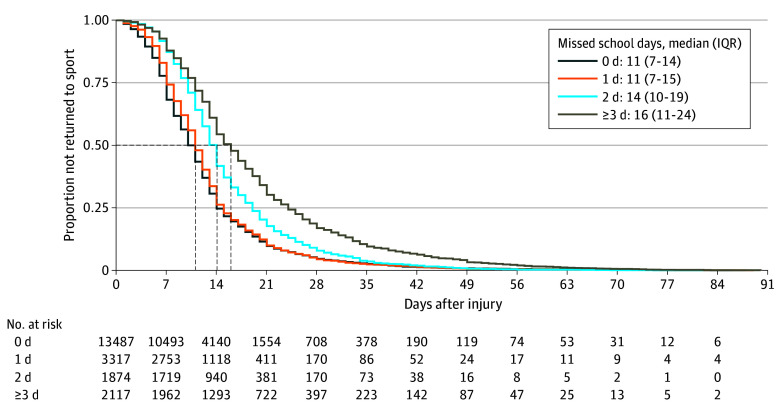
Time Missed From School in Boys and Girls Following Sport-Related Concussion Dashed lines indicate median survival time for each missed school days group.

Overall, athletes with SRC were given authorized clearance to return to full participation in a median (IQR) of 11 (7-16) days. We observed a significant association between missed school days and time to return to sport (Figure 2). The estimated mean days to return to sport were 12.15 (95% CI, 12.00-12.30) for 0 missed school days, 12.68 (95% CI, 12.39-12.96) for 1 missed school day, and 15.47 (95% CI, 15.06-15.87) for 2 missed school days. Our final multivariable Poisson regression (Table 2) revealed a significant mean 4.0% (incidence rate ratio [IRR], 1.04; 95% CI, 1.02-1.07) increase in number of days return to sport for 1 missed school day, a 27% (IRR, 1.27; 95% CI, 1.24-1.31) increase for 2 missed school days, and a 57% (IRR, 1.57; 95% CI, 1.52-1.62) increase for 3 or more missed school days relative to no missed school days. We present the estimated mean days to return to sport in Table 3, where missing 3 or more school days exhibited the highest mean time to return to sport (19.08 days; 95% CI, 18.55-19.62).

**Table 2.** Testing the Association Between Missed School Days and Time to Unrestricted Clearance With Poisson Regression and Robust SEs

Factor	Incidence rate ratio (95% CI)
Univariate	
Days absent	
0	1 [Reference]
1	1.05 (1.02-1.08)
2	1.28 (1.24-1.31)
3	1.57 (1.52-1.62)
Multivariable	
Days absent	
0	1 [Reference]
1	1.04 (1.02-1.07)
2	1.27 (1.24-1.31)
3	1.57 (1.52-1.62)
Boys	0.95 (0.93-0.97)
Season	
2015-2016	1 [Reference]
2016-2017	1.07 (1.03-1.1)
2017-2018	1.09 (1.05-1.13)
2018-2019	1.14 (1.1-1.18)
2019-2020	1.09 (1.05-1.13)
2020-2021	1.05 (1-1.1)
2021-2022	1.11 (1.07-1.16)
2022-2023	1.08 (1.05-1.12)
Concussion history	
0	1 [Reference]
1	1.09 (1.06-1.12)
2	1.16 (1.1-1.22)
Sport level	
Varsity	1 [Reference]
Middle school	0.88 (0.77-1.01)
Freshman	1.06 (1.03-1.09)
Junior varsity	1.05 (1.03-1.07)
Contact level	
Collision/contact	1 [Reference]
Limited	0.85 (0.83-0.88)
Noncontact	1.03 (0.96-1.1)
First evaluation by athletic trainer	
Yes	0.94 (0.92-0.97)

**Table 3.** Estimated Mean Days to Return to Unconditional Sport at Missed School Days from Multivariable Poisson Regression

Missed school days	Estimated mean days to return, No. (95% CI)
Overall	13.24 (13.11-13.36)
0	12.15 (12.00-12.30)
1	12.68 (12.29-12.96)
2	15.47 (15.06-15.87)
≥3	19.08 (18.55-19.62)

## Discussion

In this large retrospective cohort study, 15.9% of high school athletes missed 1 day, 9.0% missed 2 days, and 10.2% missed 3 or more school days. Moreover, return-to-sport time was slower with each additional missed school day while adjusting for additional factors previously determined to be associated with our return to sport outcome. Our findings suggest gender was significantly associated with missed school days, as 59.0% of girls and 67.9% of boys missed 0 school days after SRC. Importantly we also identified academic year, participation level (eg, varsity), contact level, competition, and having an athletic trainer as independently associated with missed school days. These findings highlight the multifaceted nature of concussion recovery, highlighting the importance of considering both athletic and academic factors when managing return-to-activity protocols for high school athletes.

Considering our statewide surveillance and large sample size, the number of days of school missed due to SRC may be generalizable to many high school athletes. Notably, this estimate aligns with the current consensus statement recommendation of 24 to 48 hours of rest before gradually returning to school.^4^ Importantly, we identified approximately 10% of athletes missed 3 or more school days due to SRC; thus, over 2000 athletes during the 8-year study period missed more than the recommended rest period.^4^ Of these athletes who missed 3 or more days, the estimated average time to return to sport was 19.08 days (95% CI, 18.55-19.62), which was significantly slower relative to those with 0 missed school days. This longer recovery time could be attributed to athletes experiencing a greater number and severity of symptoms,^4^ causing them to miss more school. Additionally, many advocate for initial return to school or work activities before returning to full sport or athletic competition, especially in pediatric populations,^4,29,30^ which may also explain our results. From our findings and other conclusions from RTL studies,^9,31^ future research should include a wider variety of RTL timelines, additional support recommendations, and accommodations for athletes based on differences in symptom number and severity following SRC. Future research should also examine if missed days could be attributed to conservative or aggressive approaches by the family independent of medical recommendations (eg, my kid is going to stay home from school until they are fully recovered vs tell them they are fine and to get back on the field).

Our study revealed that athletes returned to school earlier than the RTL timelines reported in a meta-analysis by Putukian et al,^8^ which averaged 8 days for RTL. However, there are certain differences in method that may account for discrepancies in findings. The meta-analysis defined RTL as the time it took for athletes to return to preinjury academics without any accommodations or support.^8^ In our study, we solely examined days missed from school and did not examine the duration of accommodations and RTL process. Additionally, about half of the studies included within the meta-analysis focused on collegiate and youth athletes, while our study focused inclusively on high school athletes participating within a statewide organized athletics program. Differences in academic expectations postinjury between high school and collegiate athletes, such as maintaining academic standings to retain scholarships in collegiate athletes, could contribute to differences in missed school durations.^32^ Moreover, other research recruited some athletes from specialty clinics or emergency departments,^10,11^ compared with our study, which included athletes predominantly treated by primary care physicians.^24^ Some studies have found that time to presentation is longer in specialty clinics,^33^ and symptoms or functional impairments may be more severe in these cases, leading to potential differences in where athletes seek care, which could lead to more missed school days.

As previously reported in other research,^21^ the majority of both boys and girls missed between 0 and 2 days of school following their SRC. This may be due to consensus statements^4^ recommending both boys and girls return to school within 24 to 48 hours. In contrast, Kostyun and colleagues^34^ reported girls with concussions were prescribed 3 times more academic accommodations compared with boys. The current study could not identify if high school athletes returned to school with academic accommodations, thus there could have been differences between boys and girls in the type of academic accommodation such as returning to half school days or extended time on tests. Further research is needed to determine if boys and girls are prescribed different academic accommodations and the personnel that are responsible for implementing these accommodations.

We found several additional factors independently associated with high school athletes’ number of missed school days following SRC, including season, gender, event type, sport participation level, contact level, and nonathletic trainer evaluation. The 2017 consensus statement noted limited evidence on the best rest duration for recovery with limited recommendations on return to school,^35^ so a lack of concrete guidance may have contributed to the association between academic year and missed school. Additionally, shifting to online learning during the COVID-19 pandemic could possibly be associated with a culture shift in absenteeism in more recent years.^36^ Separately, we found an association between the level of play and missed school. The intensity of play at higher levels may increase the number and severity of SRC-related head impacts,^36^ potentially explaining the association between missed school days and varsity, JV, freshman, and middle school athletes, though this could not be studied in the current database. Future research should elucidate if SRCs vary by sport level and are associated with school absence and SRC recovery trajectories. We also found an association between missed school and event type. It is possible SRCs sustained during games could have resulted in greater intensity of signs and symptoms compared with practice^15^; however, this could not be confirmed in the current analyses. Furthermore, we found that athletes not receiving their initial evaluation from an athletic trainer was associated with missed school days. Bretzin and colleagues^23^ found that athletes who were removed immediately following SRC had lower odds of prolonged (>21 days) return-to-sport when an athletic trainer was present for their initial SRC,^22^ suggesting earlier SRC management is influential to SRC recovery. Furthermore, an athletic trainer can recommend that the athlete start integrating school within the first 24 to 28 hours to aid their recovery. Advancements in research in active SRC treatment options^37,38,39,40^ are motivating many to agree that a subsymptom threshold in safe and progressive cognitive and physical activities is associated with shorter recovery timelines.^4,29,30,41^

### Limitations

This study is not without limitations. First, the MHSAA surveillance program only collects data on missed school days and does not specify if the athlete with concussion returned to school with or without academic accommodation or support. Furthermore, this study is purely observational; thus, we do not know if the athlete experienced difficulties in school during RTL. Second, we did not have access to the athletes’ symptom severity, which could have contributed to missing more school days. Furthermore, we did not assess the impact of sustaining a SRC close to a weekend on missed school days. However, experiencing an SRC before the beginning of a weekend or holiday (such as Friday) might naturally result in a break from school. Also, while this study had a large number of SRC cases, the sample was limited to only Michigan high school athletes and cannot be generalized to the rest of the US or collegiate or youth athletes. Furthermore, despite these findings representing a statewide organized athletics program, these data are deidentified, limiting us to determine key social determinants of health that may influence recovery trajectories (eg, school size, athletic trainer access, school-specific academic resources, distance from and access to a health care clinician, and socioeconomic status), which may influence the interpretation of these results. Similarly, due to deidentified data, we could not adjust for clustering by school. Thus, there is a need for continued and purposeful research that can account for these variables.

## Conclusion

This large high school surveillance program revealed athletes often missed less than 3 days of school after SRC, aligning with current consensus guidelines. However, over 2000 athletes during this 8-year study period missed 3 or more school days. While adjusting for important independent factors previously suggested to be associated with SRC recovery (ie, gender, academic year of the season, concussion history, sport participation level, contact level, and nonathletic trainer evaluation) more missed school days was associated with a slower return to unconditional sport. The result that can be communicated to key stakeholders (eg, athletes, parents, coaches, and administrators) is that an initial rest period from school of 24 to 48 hours was not detrimental to SRC return to play or recovery.

## Notes

Supplement.
Data Sharing Statement

## References

[1] Bryan MA, Rowhani-Rahbar A, Comstock RD, Rivara F; Seattle Sports Concussion Research Collaborative. Sports-and recreation-related concussions in US youth. Pediatrics. 2016;138(1):e20154635. doi:10.1542/peds.2015-4635 27325635

[2] Baldwin GT, Breiding MJ, Dawn Comstock R. Epidemiology of sports concussion in the United States. In: Hainline B, Stern RA, eds. Handbook of Clinical Neurology. Elsevier; 2018:63-74. 10.1016/B978-0-444-63954-7.00007-0 30482376

[3] Delaney JS, Lacroix VJ, Leclerc S, Johnston KM. Concussions among university football and soccer players. Clin J Sport Med. 2002;12(6):331-338. doi:10.1097/00042752-200211000-00003 12466687

[4] Patricios JS, Schneider KJ, Dvorak J, . Consensus statement on concussion in sport: the 6th International Conference on Concussion in Sport-Amsterdam, October 2022. Br J Sports Med. 2023;57(11):695-711. doi:10.1136/bjsports-2023-106898 37316210

[5] Brown NJ, Mannix RC, O’Brien MJ, Gostine D, Collins MW, Meehan WP III. Effect of cognitive activity level on duration of post-concussion symptoms. Pediatrics. 2014;133(2):e299-e304. doi:10.1542/peds.2013-2125 24394679 PMC3904277

[6] Thomas DG, Apps JN, Hoffmann RG, McCrea M, Hammeke T. Benefits of strict rest after acute concussion: a randomized controlled trial. Pediatrics. 2015;135(2):213-223. doi:10.1542/peds.2014-0966 25560444

[7] McCrea M, Broglio S, McAllister T, ; CARE Consortium Investigators. Return to play and risk of repeat concussion in collegiate football players: comparative analysis from the NCAA Concussion Study (1999-2001) and CARE Consortium (2014-2017). Br J Sports Med. 2020;54(2):102-109. doi:10.1136/bjsports-2019-100579 31036562

[8] Putukian M, Purcell L, Schneider KJ, . Clinical recovery from concussion-return to school and sport: a systematic review and meta-analysis. Br J Sports Med. 2023;57(12):798-809. doi:10.1136/bjsports-2022-106682 37316183

[9] Russell K, Selci E, Chu S, Rozbacher A, Ellis M. Academic outcomes and accommodations following adolescent sport-related concussion: a pilot study. Concussion. 2017;2(4):CNC51. doi:10.2217/cnc-2017-0009 30202592 PMC6122692

[10] Wasserman EB, Bazarian JJ, Mapstone M, Block R, van Wijngaarden E. Academic dysfunction after a concussion among US high school and college students. Am J Public Health. 2016;106(7):1247-1253. doi:10.2105/AJPH.2016.303154 27196651 PMC4984778

[11] Grubenhoff JA, Deakyne SJ, Comstock RD, Kirkwood MW, Bajaj L. Outpatient follow-up and return to school after emergency department evaluation among children with persistent post-concussion symptoms. Brain Inj. 2015;29(10):1186-1191. doi:10.3109/02699052.2015.1035325 26004755 PMC4766056

[12] Vaughan CG, Ledoux AA, Sady MD, ; PERC 5P Concussion Team. Association between early return to school following acute concussion and symptom burden at 2 weeks postinjury. JAMA Netw Open. 2023;6(1):e2251839. doi:10.1001/jamanetworkopen.2022.51839 36662524 PMC9860528

[13] Taubman B, Rosen F, McHugh J, Grady MF, Elci OU. The timing of cognitive and physical rest and recovery in concussion. J Child Neurol. 2016;31(14):1555-1560. doi:10.1177/0883073816664835 27581848

[14] O’Connor KL, Baker MM, Dalton SL, Dompier TP, Broglio SP, Kerr ZY. Epidemiology of sport-related concussions in high school athletes: National Athletic Treatment, Injury and Outcomes Network (NATION), 2011–2012 through 2013–2014. J Athl Train. 2017;52(3):175-185. doi:10.4085/1062-6050-52.1.15 28387555 PMC5384816

[15] Zuckerman SL, Kerr ZY, Yengo-Kahn A, Wasserman E, Covassin T, Solomon GS. Epidemiology of sports-related concussion in NCAA athletes from 2009-2010 to 2013-2014: incidence, recurrence, and mechanisms. Am J Sports Med. 2015;43(11):2654-2662. doi:10.1177/0363546515599634 26330572

[16] Putukian M, D’Alonzo BA, Campbell-McGovern CS, Wiebe DJ. The ivy league–big ten epidemiology of concussion study: a report on methods and first findings. Am J Sports Med. 2019;47(5):1236-1247. doi:10.1177/0363546519830100 30943078

[17] Master CL, Katz BP, Arbogast KB, ; CARE Consortium Investigators. Differences in sport-related concussion for female and male athletes in comparable collegiate sports: a study from the NCAA-DoD Concussion Assessment, Research and Education (CARE) Consortium. Br J Sports Med. 2021;55(24):1387-1394. doi:10.1136/bjsports-2020-103316 33355211

[18] Brett BL, Breedlove K, McAllister TW, ; CARE Consortium Investigators. Investigating the range of symptom endorsement at initiation of a graduated return-to-play protocol after concussion and duration of the protocol: a study from the National Collegiate Athletic Association–Department of Defense Concussion, Assessment, Research, and Education (CARE) Consortium. Am J Sports Med. 2020;48(6):1476-1484. doi:10.1177/0363546520913252 32298132

[19] Churchill NW, Hutchison MG, Graham SJ, Schweizer TA. Sex differences in acute and long-term brain recovery after concussion. Hum Brain Mapp. 2021;42(18):5814-5826. doi:10.1002/hbm.25591 34643005 PMC8596946

[20] Frommer LJ, Gurka KK, Cross KM, Ingersoll CD, Comstock RD, Saliba SA. Sex differences in concussion symptoms of high school athletes. J Athl Train. 2011;46(1):76-84. doi:10.4085/1062-6050-46.1.76 21214354 PMC3017493

[21] Bretzin AC, Covassin T, Fox ME, . Sex differences in the clinical incidence of concussions, missed school days, and time loss in high school student-athletes: part 1. Am J Sports Med. 2018;46(9):2263-2269. doi:10.1177/0363546518778251 29879362

[22] Bretzin AC, Esopenko C, D’Alonzo BA, Wiebe DJ. Clinical recovery timelines after sport-related concussion in men’s and women’s collegiate sports. J Athl Train. 2022;57(7):678-687. doi:10.4085/601-20 33626145 PMC9528707

[23] Bretzin AC, Zynda AJ, Pollard-McGrandy AM, Wiebe DJ, Covassin T. Acute sport-related concussion management and return to sport time in high school athletes. Am J Sports Med. 2024;52(3):791-800. doi:10.1177/03635465231219263 38279802

[24] Bretzin AC, Zynda AJ, Wiebe DJ, Covassin T. Time to authorized clearance from sport-related concussion: the influence of health care provider and medical facility. J Athl Train. 2021;56(8):869-878. doi:10.4085/JAT0159-20 33351918 PMC8359712

[25] Zynda AJ, Petit KM, Anderson M, Tomczyk CP, Covassin T. Removal from activity after sports-related concussion in sex-comparable sports from the Michigan High School Athletic Association. Am J Sports Med. 2021;49(10):2810-2816. doi:10.1177/03635465211020007 34181487

[26] Field N, Cohen T, Struelens MJ, . Strengthening the Reporting of Molecular Epidemiology for Infectious Diseases (STROME-ID): an extension of the STROBE statement. Lancet Infect Dis. 2014;14(4):341-352. doi:10.1016/S1473-3099(13)70324-4 24631223

[27] Rice SG; American Academy of Pediatrics Council on Sports Medicine and Fitness. Medical conditions affecting sports participation. Pediatrics. 2008;121(4):841-848. doi:10.1542/peds.2008-0080 18381550

[28] Michigan High School Athletic Association. Return to activity and post-concussion consent form. 2023. Accessed September 11, 2023. https://my.mhsaa.com/portals/0/documents/health%20safety/1617returntoplay.pdf

[29] Broglio S, Register-Mihalik J, Guskiewicz K, Leddy J, Merriman A, Valovich McLeod T. National athletic trainers’ association bridge statement: management of sport-related concussion. J Athl Train. 2024;59:225-242. doi:10.4085/1062-6050-0046.22 38530653 PMC10976337

[30] Davis GA, Schneider KJ, Anderson V, . Pediatric sport-related concussion: recommendations from the Amsterdam Consensus Statement 2023. Pediatrics. 2024;153(1):e2023063489. doi:10.1542/peds.2023-063489 38044802

[31] Baker JG, Leddy JJ, Darling SR, . Factors associated with problems for adolescents returning to the classroom after sport-related concussion. Clin Pediatr (Phila). 2015;54(10):961-968. doi:10.1177/0009922815588820 26084537

[32] Holmes A, Chen Z, Yahng L, Fletcher D, Kawata K. Return to learn: academic effects of concussion in high school and college student-athletes. Front Pediatr. 2020;8:57. doi:10.3389/fped.2020.00057 32195210 PMC7065268

[33] Taubman B, Michael Luciani A, Gealt DB, Drake TP, Cochetti P, Farrar JT. The care of the concussed pediatric patient prior to presentation to primary care pediatrician versus concussion specialists: implications for management. J Concussion. 2021;5:2059700221998921. doi:10.1177/2059700221998921

[34] Kostyun RO, Hafeez I. Protracted recovery from a concussion: a focus on gender and treatment interventions in an adolescent population. Sports Health. 2015;7(1):52-57. doi:10.1177/1941738114555075 25553213 PMC4272696

[35] McCrory P, Meeuwisse W, Dvořák J, . Consensus statement on concussion in sport-the 5th international conference on concussion in sport held in Berlin, October 2016. Br J Sports Med. 2017;51(11):838-847. doi:10.1136/bjsports-2017-097699 28446457

[36] Kundu A, Bej T. COVID-19 response: students’ readiness for shifting classes online. Int J Bus Soc. 2021;21(6):1250-1270.

[37] Haider MN, Cole WR, Willer BS, . Early targeted heart rate exercise is safe and may hasten return-to-duty in service members with acute concussion, a preliminary study. Brain Inj. 2024;38(2):119-125. doi:10.1080/02699052.2024.2306334 38329063

[38] Leddy JJ, Burma JS, Toomey CM, . Rest and exercise early after sport-related concussion: a systematic review and meta-analysis. Br J Sports Med. 2023;57(12):762-770. doi:10.1136/bjsports-2022-106676 37316185

[39] Sinnott AM, Kochick VL, Eagle SR, . Comparison of physiological outcomes after dynamic exertion between athletes at return to sport from concussion and controls: preliminary findings. J Sci Med Sport. 2023;26(12):682-687. doi:10.1016/j.jsams.2023.09.014 37793956

[40] Kontos AP, Eagle SR, Braithwaite R, . The effects of rest on concussion symptom resolution and recovery time: a meta-analytic review and subgroup analysis of 4329 patients. Am J Sports Med. 2023;51(14):3893-3903. doi:10.1177/03635465221150214 36847271

[41] Wiebe D, Bretzin A, D’Alonzo B. Progression through return-to-sport and return-to-academics guidelines for concussion management and recovery in collegiate student athletes: findings from the Ivy League–Big Ten Epidemiology of Concussion Study. Br J Sports Med. 2022;56(14):801-811. doi:10.1136/bjsports-2021-104451 35444018 PMC9252856

