# Supplementary material for: Missing School Days Following Sport-Related Concussion in High School Athletes
Source: JAMA Netw Open. 2024 Oct 18;7(10):e2440264. doi: 10.1001/jamanetworkopen.2024.40264 (PMC11581575; doi:10.1001/jamanetworkopen.2024.40264)
Supplement: Supplement. — Data Sharing Statement [file jamanetwopen-e2440264-s001.pdf]

Data Sharing Statement

Covassin. Missing School Days Following Sports-Related Concussion in High School Athletes. *JAMA Netw Open*. Published October 18, 2024. doi:10.1001/jamanetworkopen.2024.40264

Data
Data available: No
